# Tissue-infiltrating lymphocytes signature predicts survival in patients with early/intermediate stage hepatocellular carcinoma

**DOI:** 10.1186/s12916-019-1341-6

**Published:** 2019-06-05

**Authors:** Meng-Xin Tian, Wei-Ren Liu, Han Wang, Yu-Fu Zhou, Lei Jin, Xi-Fei Jiang, Chen-Yang Tao, Zheng Tang, Pei-Yun Zhou, Yuan Fang, Wei-Feng Qu, Zhen-Bin Ding, Yuan-Fei Peng, Zhi Dai, Shuang-Jian Qiu, Jian Zhou, Wan Yee Lau, Jia Fan, Ying-Hong Shi

**Affiliations:** 1 Department of Liver Surgery, Key Laboratory of Carcinogenesis and Cancer Invasion of Ministry of Education, Liver Cancer Institute, Zhongshan Hospital, Fudan University, 180 Fenglin Road, Shanghai, 200032 People’s Republic of China; 2 Institutes of Biomedical Sciences, Fudan University, Shanghai, People’s Republic of China; 3 Faculty of Medicine, the Chinese University of Hong Kong, Shatin, New Territories, Hong Kong, SAR China

**Keywords:** Hepatocellular carcinoma, Survival prediction, Immune signature, Prognosis

## Abstract

**Background:**

Intratumoral immune infiltrates have manifested a robust prognostic signature in patients with hepatocellular carcinoma (HCC). We hypothesized that a novel tissue-related immune signature (TRIS) could improve the prediction of postoperative survival for patients diagnosed with early/intermediate HCC.

**Methods:**

Twenty-eight immune features were immunohistochemically examined on 352 HCC specimens. The LASSO Cox regression model was used to construct a five-feature-based TRIS. The univariate and multivariate Cox analyses were performed. Based on independent predictors, the immune-clinical prognostic index (ICPI) was established. Performance assessment was measured with C-index and compared with seven traditional staging systems. The independent validation cohort (*n* = 393) was included to validate the model.

**Results:**

By using the LASSO method, the TRIS were constructed on the basis of five immune features, CD3_intratumoral (T)_, CD27_T_, CD68_peritumoral (P)_, CD103_T_, and PD1_T_. Multivariate Cox analysis showed that the TRIS was an independent prognostic predictor. In the training cohort, γ-glutamyl transferase, tumor diameter, tumor differentiation, and TRIS were incorporated into the ICPI. The ICPI presented satisfactory discrimination ability, with C-index values of 0.691 and 0.686 in the training and validation cohorts, respectively. Compared with seven conventional staging systems (C-index, training cohort, 0.548–0.597; validation cohort, 0.519–0.610), the ICPI exhibited better performance for early/intermediate-stage HCCs. Further, the patients were categorized into three subgroups with X-tile software, and the stratified ICPI presented a superior corrected Akaike information criterion and homogeneity in both cohorts.

**Conclusions:**

Our ICPI was a useful and reliable prognostic tool which may offer good individualized prediction capability for HCC patients with early/intermediate stage.

**Electronic supplementary material:**

The online version of this article (10.1186/s12916-019-1341-6) contains supplementary material, which is available to authorized users.

## Background

Hepatocellular carcinoma (HCC) is a leading cause of cancer-related death worldwide, accounting for more than 700,000 deaths per year [1]. The prognosis of HCC not only depends on the tumor burden of patients but also on their underlying liver functional reserve [2] and tumor heterogeneity [3]. Therefore, in order to promote better prognosis and reduce the global burden of this disease, it is crucial to identify new pathological and biological predictors and improve prediction of postsurgical survival for HCC.

While multiple immune components are involved in cancer initiation and progression [4], the existence of various immune components have been identified in the liver, including Kupffer cells [5], dendritic cells (DCs) [6], natural killer (NK) cells, naïve and memory lymphocytes [7], B cells [8], T regulatory cells (Tregs) [9], T follicular helper (Tfh) cells [10], CD8^+^ T cells [11], and CD4^+^ T cells [12]. Studies have suggested that immune infiltrates are of clinical significance in various types of cancer [13–15]. For example, the immune score, which is based on the number of lymphocytic populations in the tumor core and the invasive margin of tumor, was able to indicate outcomes in patients with early-stage colorectal cancer [16] and has been defined as a new component in the classification of colorectal cancer. In our previous studies, intratumoral neutrophils [17], margin-infiltrating CD20^+^ B cells by Shi et al. [18], and intratumoral balance of regulatory and cytotoxic T cells by Gao et al. [19] were found to be associated with long-term survival of HCC patients. It was reported that about 25% of HCC samples were found expressing markers of an inflammatory response, characterized with high expression levels of programmed cell death protein 1 (PD1) and CD274, and markers of cytolytic activity [20]. Li et al. have also reported that the expression of PD1 in HCC was able to promote tumor growth independent of adaptive immunity [21]. However, the prognostic potential of infiltrating immune cells in survival prediction for patients with HCC has not yet been assessed comprehensively.

In this study, we used the least absolute shrinkage and selection operator (LASSO) regression model based on 28 immunological features to establish the tissue-related immune signature (TRIS). Then, independent clinicopathological predictors and TRIS were integrated into a novel immune-clinical prognostic index (ICPI). Moreover, we investigated the performance of the ICPI, compared the ICPI with 7 conventional staging systems, and validated the ICPI model in the validation cohort.

## Methods

### Dataset

Data collection was conducted from all HCC patients who underwent liver resection between April 2005 and September 2008 at the Department of Liver Surgery, Zhongshan Hospital, Shanghai, China. The inclusion criteria were without previous anticancer therapy, absence of any other types of malignancies, complete resection of liver tumors, histopathological confirmation of HCC, and classified as stage 0 or A, or B according to the Barcelona Clinic Liver Cancer (BCLC) staging system. In this study, the patients with BCLC B stage were carefully selected: resectable HCC, adequate liver remnant size after liver resection, no radiological evidence of vascular invasion or extrahepatic metastasis, and liver function status of Child A-B. Patients with hilar or extrahepatic cholangiocarcinoma, tumors of uncertain origin, metastatic liver tumors, combined hepatocellular-cholangiocarcinoma, perioperative mortality, or tumors beyond BCLC stage B were excluded from the study. Recruited patients were divided into 2 cohorts: the training and validation cohorts. The training cohort included patients who received surgery from April 2005 to December 2006. The validation cohort consisted of patients that received surgery from January 2007 to September 2008. The flowchart of patient recruitment and group assignment is presented in Additional file 3: Figure S1.

### Follow-up

The study was censored on December 1, 2011. A standardized follow-up protocol was adopted for all patients [22]. The patients attended follow-up visits with computed tomography or abdominal magnetic resonance imaging scans carried out once every 6 months for the first 2 years. Abdominal ultrasound, liver function tests, and serum alpha fetoprotein (AFP) level examinations were performed once every 3 months. The endpoints of the study were overall survival (OS) and recurrence-free survival (RFS). OS was defined as the interval between the date of surgery and the date of patient death or the last follow-up visit. RFS was defined as the time from the date of tumor resection to the date of diagnosis of recurrence/metastasis, or the last follow-up visit.

### Tissue microarray (TMA) construction

All HCC tumor specimens were examined independently by two reviewers who were blinded to the clinical characteristics or treatment outcomes of patients. The TMA construction was conducted as previously described [19]. To ensure homogeneity and reproducibility, two representative areas with infiltrating lymphocytes were carefully selected by qualified pathologists in H&E-stained slides of the blocks from the tumor center and peritumor tissues. Then, duplicate cylinders (1 mm in diameter) from two different areas were punched, arrayed, and re-embedded in a recipient block. All final slides were dipped in paraffin for preservation and stored at 4 °C before immunohistochemistry (IHC) assays.

In this study, immune biomarkers were selected for IHC staining based on their close involvement in patient survival and tumor recurrence (CD3, CD8, CD4, CD57, and CD68) [17, 18, 23–26], local immune response (CD20, CD27, and CD45RO) [8, 27], tumor growth (PD1 and CD66b) [21, 28], and antitumor function (CD14) [29]. CXCR5 was selected because of its participation in the pathogenesis of primary biliary cirrhosis [30]. While the prognostic values of CD45RA [31] and CD103 [32] in liver cancer still remain unclear, they were also selected due to their presence in tumors. The details regarding IHC, antibodies, and staining conditions are included in the Additional file 1: Supplementary methods.

### Evaluation of immunohistochemical staining

To evaluate the tissue-infiltrating immune cells, the tissue sections were screened at low power (× 100), and the 5 most representative and independent fields were selected using the Leica Qwin Plus v3 software to ensure representativeness and homogeneity. The respective areas of the nontumoral and intratumoral regions were then photographed at × 200 magnification. Identical settings were used for each photograph. High-resolution spot images (1360 × 1024) were obtained and stored under TIFF format. All the consecutive images were analyzed using a computer-automated method (Image-pro plus 6.0, Media Cybernetics Inc.) as described elsewhere [33]. The spot and captured spot (× 200) with image software are presented in Additional file 3: Figure S2. The numbers of positively stained cells were recorded, and the mean value was used for statistical analysis. The 5 representative spots showed a good level of homogeneity of stained cell numbers in tumor or non-tumor regions.

### Statistical analysis

Summary statistics were obtained using established methods and presented as percentages or median values. Pearson’s *χ*^2^ test or Fisher’s exact test was employed to compare categorical variables. Wilcoxon rank sum test or Student’s *t* test was used to evaluate continuous variables. The OS was estimated by the Kaplan-Meier method and compared with the log-rank test. Cluster version 3.0 (Michiel de Hoon, Tokyo, Japan) was performed for the hierarchical clustering of multi-immune features [34]. The estimation of the relative fractions of immune cells from tissue expression profiles of HCC was conducted using CIBERSORT [35]. The details regarding CIBERSORT and construction of immune network are included in Additional file 1: Supplementary methods.

LASSO is a broadly used method for regression with high-dimensional predictors [36]. We applied the LASSO Cox analysis to identify significant prognostic immune features and constructed a multi-immune feature (TRIS score) on the basis of OS. The “glment” package was used to do the LASSO Cox analysis. By using the univariate and multivariate Cox proportional hazards regression in the training dataset, we integrated independent prognostic factors into the ICPI model. The nomogram and calibration plots were constructed as previously described [37]. We compared the ICPI model with American Joint Committee on Cancer (AJCC) 7th edition, AJCC 8th edition, Cancer of the Liver Italian Program (CLIP), Barcelona Clínic Liver Cancer (BCLC), Okuda, Japan Integrated Staging (JIS) and Liver Cancer Study Group of Japan (LCSGJ) staging systems based on receiver operating characteristic (ROC) curves. The *P* value for the c-indices in the 2 models was computed using a bootstrapping method [38]. The rcorrp.cens package in Hmisc was used.

X-tile software was used to generate the optimum cutoff point for continuous variables according to the highest *χ*^2^ value defined by the Kaplan-Meier survival analysis and the log-rank test [39]. Based on the ICPI score, HCC patients were categorized into 3 subgroups with X-tile software version 3.6.1 (Yale University School of Medicine, New Haven, CT, USA). To evaluate the performance of stratified ICPI and other staging systems, the corrected Akaike information criterion (AIC) was chosen to present how the staging system correlated with patient survival. And then, Wald’s *χ*^2^ test was used to evaluate homogeneity in the same stage within each system, indicating differences in survival among patients [40]. Statistical analyses were performed with R software version 3.1.0 (R Foundation for Statistical Computing, Vienna, Austria). Statistical significance was set at 0.05.

## Results

### Clinicopathological characteristics of patients

The demographic and clinicopathological data of the 745 HCC patients recruited in this study are shown in Table 1. The patients were divided into the training cohort (*n* = 352) and the validation cohort (*n* = 393). The percentages of patients at BCLC stages 0, A, and B in the training cohort were 11.9%, 76.4%, and 11.7%, respectively. Except for albumin and bilirubin, no significant differences were observed between the training and validation cohorts in any other patient, tumor, or operation-related covariates. Despite significant differences existed in the albumin and bilirubin levels between the two cohorts, these two indicators were still within the normal range and would not influence the liver function of HCC patients.

**Table 1:** Demographic, clinical, and tumor characteristics of patients with hepatocellular carcinoma

Patient demographics	Training cohort (*n* = 352)	Validation cohort (*n* = 393)	*P* value
Age, years			
< 60	274 (77.8%)	290 (73.8%)	0.20
≥ 60	78 (22.2%)	103 (26.2%)	
Sex (female), *n* (%)	60 (17.0%)	53 (13.5%)	0.17
Etiology			
HBV	295 (83.8%)	314 (79.9%)	0.24
HCV	2 (0.6%)	6 (1.5%)	
Others	55 (15.6%)	73 18.6%)	
Liver cirrhosis, yes (%)	284 (80.7%)	331 (84.2%)	0.06
AFP, ng/mL	101.5 (6.0, 724.5)	71 (6.0, 865.0)	0.45
Albumin, g/L	4.3 (4.0, 4.6)	4.4 (4.1, 4.7)	0.008
Bilirubin, μmol/L	14.8 (11.5, 18.6)	14.0 (10.6, 18.3)	0.03
ALT, IU/L	41 (27.5, 63.5)	38 (27, 54)	0.06
GGT, U/L	52 (33, 99)	58 (38, 100)	0.11
Tumor number, *n* (%)			
1	314 (89.2%)	327 (83.2%)	0.06
2	29 (8.2%)	51 (14.5%)	
≥ 3	9 (2.6%)	15 (3.8%)	
Tumor diameter, cm	4.0 (2.5, 7.0)	4.0 (2.5, 6.5)	0.39
Microvascular invasion (yes), *n* (%)	111 (31.5%)	114 (29.0%)	0.45
Lymphoid metastasis (negative), *n* (%)	350 (99.4%)	393 (100.0%)	0.13
Tumor differentiation (Edmondson-Steiner grade)			
I-II	266 (75.6%)	284 (72.3%)	0.31
III-IV	86 (24.4%)	109 (27.7%)	
BCLC			
0	42 (11.9%)	46 (11.7%)	0.52
A	269 (76.4%)	311 (79.1%)	
B	41 (11.7%)	36 (9.2%)	
Occlusion, min			
< 15	274 (77.8%)	299 (76.1%)	0.57
≥ 15	78 (22.2%)	94 (23.9%)	

Values are presented as no. (%) or median (Q1, Q3)

*HBV* hepatitis B virus, *HCV* hepatitis C virus, *AFP* α-fetoprotein, *ALT* alanine aminotransferase, *GGT* γ-glutamyl transferase

After a median follow-up of 52.2 months (range, 3.0 to 79.3) for the entire study population, 54.8% of patients (408/745) had developed tumor recurrence, and 38.3% (285/745) had died. The 1-, 3-, and 5-year OS rates were 88.9%, 69.7%, and 56.3%, respectively, and the 1-, 3-, and 5-year RFS rates were 73.4%, 54.0%, and 36.5%, respectively.

### Immune characteristics of HCC tissues

**Fig. 1:**
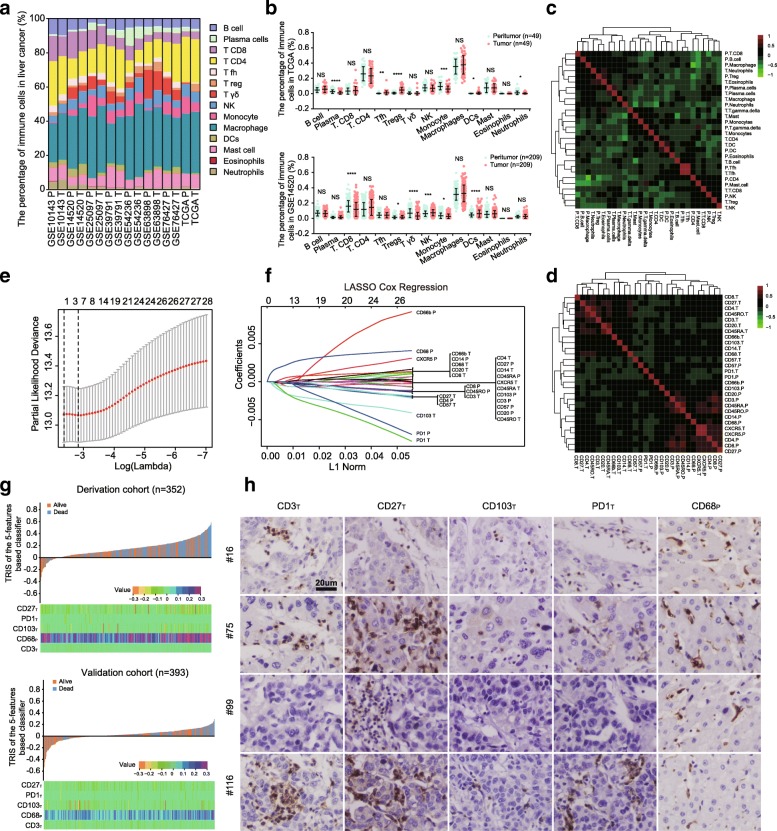
Characteristics of the immune microenvironment and selection of immune features by LASSO analysis in liver cancer. **a** Relative fractions of 22 leukocyte subsets across 8 datasets estimated by CIBERSORT. **b** Comparison of immune cells between neoplastic and adjacent tissues in the GSE14520 and TCGA datasets. *, **, ***, and **** denote *P* < .05, < 0.01, < 0.001, and < 0.0001, respectively. NS denotes no significance (Student’s *t* test). **c**, **d** Correlation matrix followed by unsupervised hierarchical clustering in immune cell fractions of TCGA (**c**) and 28 immune features of HCC tissues (**d**). Pearson correlation coefficients (*R*) were calculated. Correlation coefficients were plotted with negative correlation (green), positive correlation (red), and *R* = 0 (black). **e**, **f** Five immune features selected by LASSO Cox regression analysis. Left panel: the two dotted vertical lines are drawn at the optimal values by minimum criteria (**e**) and 1-s.e. criteria (**f**). Right panel: LASSO coefficient profiles of the 28 immune features. **g** TRIS distribution of the 5-immune features in the training and validation cohorts. Upper panel: TRIS distribution of the five-immune-feature-based classifier and patient survival status. Lower panel: heatmap presenting density of the five immune features in HCC patients. **h** IHC expression pattern of selected immune features, including CD3_T_, CD27_T_, CD103_T_, PD1_T_, and CD68_P_ in 4 different patients. Bar, 20 μm. T is for tumor specimens and P for peritumor specimens

To investigate the cellular composition of the immune infiltrates in liver cancer, we initially built the CIBERSORT-inferred relative fractions of the different immune cell types with publicly available data (TCGA and 7 GEO datasets) [35]. Among the 8 datasets, the percentage of macrophages was the highest, followed by CD4^+^ T cells, mast cells, and CD8^+^ T cells (Fig. 1a). Student’s *t* test revealed that the percentages of plasma cell, monocyte, CD8^+^ T cell, and neutrophil contents were decreased in intratumoral tissues, while the percentages of Tfh cells, Tregs, NK cells, and DCs were increased in TCGA and GSE14520 datasets (Fig. 1b). Further, we investigated the coordination of immune cell fractions in TCGA dataset. The correlation analysis was visualized using the unsupervised hierarchical clustering of a correlation matrix of immune cell analysis [34]. Figure 1c shows 2 clusters characterized by immune cells of an exhausted immune response (neutrophils_intratumoral (T)_, eosinophils_T_, and Treg_peritumor (P)_ cells) and an adaptive T cell response (Tfh_T_ and Tfh_P_), respectively.

To confirm these results, we evaluated the density of 28 immune features in 2 regions of liver cancer using tissue microarrays: tumor and peritumor. The expression levels of 28 immune features are presented in Additional file 3: Figure S3. Based on the correlation analysis, three major clusters were identified. Two clusters were characterized with functional coordination of T lymphocytes in the intratumoral tissues (CD3_T_, CD4_T_, CD27_T_, and CD45RO_T_) or the adjacent normal tissues (CD3_P_, CD45RA_P_, and CD45RO_P_). The last cluster was characterized with the functional coordination of peri- and intratumoral CXCR5^+^ cells (a biomarker of Tfh) [30], which is consistent with the results of TCGA dataset (Tfh_T_ and Tfh_P_) (Fig. 1d). In summary, these data indicated a high degree of functional coordination of specific types of immune cells.

### Construction of the TRIS

To select prognostic immune features, we performed the LASSO Cox regression model on the basis of OS [36]. Five immune features were identified in the training cohort: CD3_T_, CD27_T_, CD103_T_, PD1_T_, and CD68_P_ (Fig. 1e–g). The IHC expression modes of the 5 immune features in HCC tissues are presented in Fig. 1h. And then, we built a personalized TRIS with the coefficients weighted by the LASSO Cox model in the training cohort, where TRIS = (175.6642 × the level of CD68_P_ − 14.7372 × the level of CD3_T_ − 37.5718 × the level of CD27_T_ − 88.1263 × the level of CD103_T_ − 50.5161 × the level of PD1_T_) × 10^−5^. The level of immune features was defined as the number of positively stained cells in specific regions of each HCC patient: intra- or peri-tumoral tissues. We then evaluated the correlation of TRIS with clinicopathological variables and observed that the TRIS was statistically associated with sex (*P* = 0.02), AFP (*P* = 0.009), tumor diameter (*P* = 0.02), and tumor numbers (*P* = 0.046) (Additional file 2: Table S1).

### Selection of prognostic predictors

Univariate analyses were performed on age, sex, etiology, TRIS, and clinicopathologic variables to determine their associations with OS in patients with early/intermediate-stage HCC. All variables that were significant were evaluated with multivariate analyses (Table 2). Multivariate analysis revealed that γ-glutamyl transferase (GGT) (hazard ratio (HR), 1.002; 95% CI, 1.000–1.004; *P* = 0.01), tumor diameter (HR, 1.100; 95% CI, 1.046–1.156; *P* < 0.001), tumor differentiation (Edmondson-Steiner grade I-II/III-IV) (HR, 1.819; 95% CI, 1.268–2.610; *P* = 0.001) [41], and TRIS (HR, 4.606; 95% CI, 1.335–15.890; *P* = 0.02) were independent prognostic factors of OS in HCC patients.

**Table 2:** Univariate and multivariate Cox analysis of OS in the training cohort (*n* = 352)

	Univariate analysis		Multivariate analysis	
	HR (95% CI)	*P* value	HR (95% CI)	*P* value
Sex (female/male)	0.934 (0.611, 1.428)	0.75		
Age, years	1.007 (0.991, 1.024)	0.38		
HBsAg (yes/no)	0.915 (0.590, 1.419)	0.69		
HBcAb (yes/no)	0.664 (0.405, 1.088)	0.12		
Anti-HCV (yes/no)	1.671 (0.234, 11.951)	0.61		
Bilirubin, μmol/L	0.987 (0.961, 1.014)	0.36		
ALT, IU/L	1.002 (1.000, 1.004)	0.12		
Albumin, g/L	0.738 (0.526, 1.035)	0.08		
AFP, ng/mL	1.000 (1.000, 1.000)	0.19		
GGT, U/L	1.002 (1.001, 1.004)	< 0.001	1.002 (1.000, 1.004)	0.01
Liver cirrhosis (yes/no)	1.461 (0.919, 2.319)	0.11		
Tumor diameter, cm	1.153 (1.107, 1.202)	< 0.001	1.100 (1.046, 1.156)	< 0.001
Tumor number	1.259 (1.004, 1.583)	0.046	1.266 (0.976, 1.643)	0.08
Microvascular invasion (yes/no)	1.362 (1.065, 1.767)	0.02	1.211 (0.995, 1.599)	0.09
Lymphoid metastasis (yes/no)	1.833 (0.256, 13.108)	0.55		
Tumor differentiation (Edmondson-Steiner grade I-II/III-IV)	1.753 (1.232, 2.494)	0.002	1.819 (1.268, 2.610)	0.001
Occlusion time, min	1.028 (1.011, 1.045)	< 0.001	1.013 (0.996, 1.031)	0.14
TRIS	12.197 (3.835, 38.791)	< 0.001	4.606 (1.335, 15.89)	0.02

*HBsAg* hepatitis B surface antigen, *HBcAb* hepatitis B core antibody, *HCV* hepatitis C virus, *AFP* α-fetoprotein, *ALT* alanine aminotransferase, *GGT* γ-glutamyl transferase, *AFP* alpha fetoprotein, *TRIS* tissue-related immune signature

### Establishment of the ICPI

To further improve the accuracy of survival prediction, GGT, TRIS, tumor diameter, and tumor differentiation were integrated. By using the Cox proportional hazards regression model, we then derived an individualized ICPI equation: (0.0889 × GGT + 5 × tumor diameter + 25.4939 × tumor differentiation status + 60.9038 × TRIS − 1.1324). In this formula, Edmondson-Steiner grade I-II was defined as 1, and grade III-IV as 2. And then, we evaluated the predictive value of ICPI with other postoperative variables. The data revealed that the performance of ICPI was superior to that of postoperative variables, including microvascular invasion, lymphoid metastasis, and tumor differentiation (Additional file 2: Table S2).

**Fig. 2:**
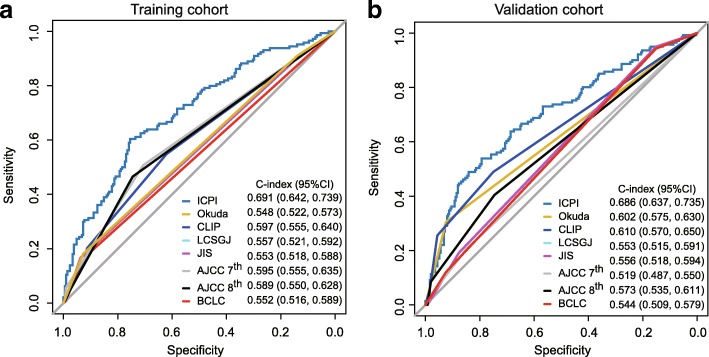
ROC curves of overall survival (OS) for the ICPI and other 7 staging systems in the training (**a**) and validation (**b**) cohorts. The C-index value of the ICPI model for OS was significantly higher than those for the Okuda, CLIP, LCSGJ, JIS, AJCC 7th edition, AJCC 8th edition, and BCLC classifications for OS [0.691 vs 0.548 (*P* < 0.001), 0.597 (*P* < 0.001), 0.557 (*P* < 0.001), 0.553 (*P* < 0.001), 0.595 (*P* = 0.008), 0.589 (*P* = 0.002), 0.552 (*P* < 0.001), respectively] in the training cohort. The validation cohort shared the similar trend [ICPI vs Okuda 0.686 vs 0.602 (*P* < 0.001), CLIP 0.610 (*P* = 0.01), LCSGJ 0.553 (*P* < 0.001), JIS 0.556 (*P* < 0.001), AJCC 7th edition 0.519 (*P* < 0.001), AJCC 8th edition 0.573 (*P* < 0.001), BCLC 0.544 (*P* < 0.001), respectively]

To compare the predictive power of the ICPI and 7 traditional staging systems, ROC curve analysis was applied. In patients with HBV-associated HCC, our established ICPI achieved significantly improved estimation in survival prediction (C-index, 0.691) when compared with the 7 staging systems in the training cohort (C-index, 0.548–0.597) (Fig. 2a and Additional file 2: Table S3).

### Performance of the ICPI in stratifying the risk of patients

To determine the optimal cutoff values of the ICPI, X-tile program [39] was used in the training cohort (Additional file 3: Figure S4). Then, the patients were stratified into 3 subgroups: score 1, 0 to 58.5; score 2, 58.5 to 86.2; and score 3, ≥ 86.2. In the training cohort, stratification into the 3 subgroups allowed significant distinction for survival outcomes (score 1 vs. 2, *P* = 0.001; score 2 vs. 3, *P* <  0.001). Except for Okuda staging system, no significant differences in survival distribution were observed across all stages of BCLC, CLIP, JIS, LCSGJ, and AJCC 7th and 8th edition staging systems **(**Additional file 3: Figure S5). Further, we assessed the performance of the ICPI model and 7 staging systems with corrected AIC values and homogeneity [40]. Among the eight staging systems, our stratified ICPI revealed the highest homogeneity (43.66) and the lowest AIC value (1594.64) in HBV-related HCC patients in the early/intermediate stage (Table 3), implying that stratified ICPI might be more accurate in predicting postoperative survival.

**Table 3:** Comparison of prognostic performance among HCC staging systems and the novel prognostic system

Model	Training cohort (n = 352)		Validation cohort (n = 393)	
	Homogeneity (Wald *χ*^2^)	Corrected AIC	Homogeneity (Wald *χ*^2^)	Corrected AIC
Stratified ICPI	43.66	1594.64	50.94	1551.86
AJCC 7th edition	20.22	1620.07	4.67	1600.13
AJCC 8th edition	17.80	1622.50	17.55	1587.25
LCSGJ	10.52	1629.78	12.57	1592.23
BCLC	9.27	1629.03	9.29	1593.50
JIS	10.77	1629.52	14.13	1590.66
Okuda	8.41	1627.88	35.67	1565.13
CLIP	18.56	1623.74	37.91	1566.89

### Validation of constructed ICPI

To substantiate the performance of ICPI, validation analyses were performed in an internal validation cohort (*n* = 393). In the validation cohort, the C-index value of the ICPI was superior than those of 7 other staging systems [ICPI vs Okuda, 0.686 vs 0.602 (*P* <  0.001); CLIP, 0.610 (*P* = 0.01); LCSGJ, 0.553 (*P* <  0.001); JIS, 0.556 (*P* < 0.001); AJCC 7th edition, 0.519 (*P* < 0.001); AJCC 8th edition, 0.573 (*P* < 0.001); BCLC, 0.544 (*P* < 0.001), respectively] (Fig. 2 and Additional file 2: Table S3).

**Fig. 3:**
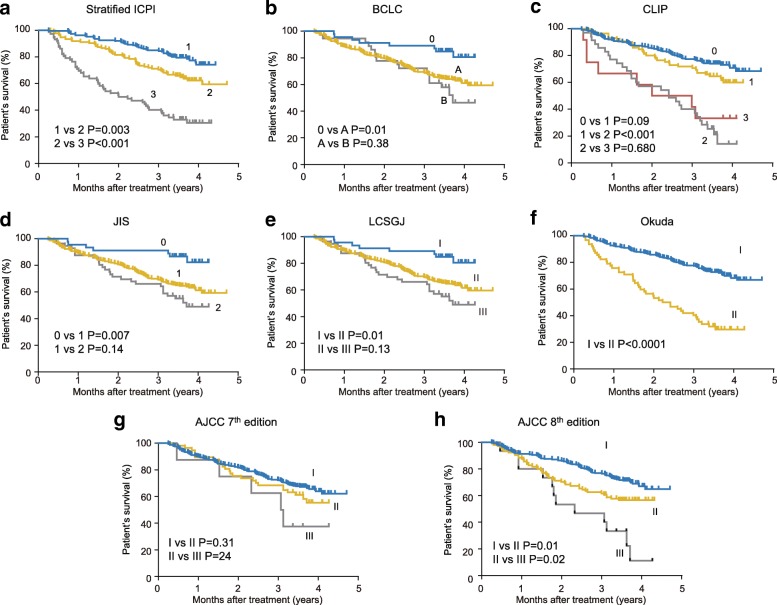
Kaplan-Meier survival curves of the validation cohort categorized by different staging systems (**a** stratified ICPI, **b** BCLC, **c** CLIP, **d** JIS, **e** LCSGJ, **f** Okuda, **g** AJCC 7th edition, **h** AJCC 8th edition)

Moreover, we performed the Kaplan-Meier survival analysis according to the stratified ICPI and traditional staging systems in the validation cohort. Figure 3 indicates that significant differences in survival distributions were found across all stages of stratified ICPI score 1/2 (*P* = 0.003), score 2/3 (*P* < 0.001), Okuda stage I/II (*P* < 0.001), and AJCC 8th edition stage I/II (*P* = 0.01) and II/III (*P* = 0.02). There were no significant survival differences between BCLC stage A/B (*P* = 0.38), CLIP 0/1 (*P* = 0.09), 2/3 (*P* = 0.68), JIS 1/2 (*P* = 0.14), LCSGJ II/III (*P* = 0.13), AJCC 7th edition I/II (*P* = 0.31), and II/III (*P* = 0.24).

We also assessed corrected AIC values and homogeneity in the validation cohort. The stratified ICPI remains the highest homogeneity (50.94) and lowest AIC (1551.86) (Table 3). In addition, we generated a nomogram to facilitate the clinical application of ICPI (Additional file 3: Figure S6A). The calibration plots achieved acceptable agreement in the validation cohort between the ICPI prediction and the actual observation for 1-, 3-, and 5-year OS (Additional file 3: Figure S6B).

### Relationship between the ICPI and local immune status

**Fig. 4:**
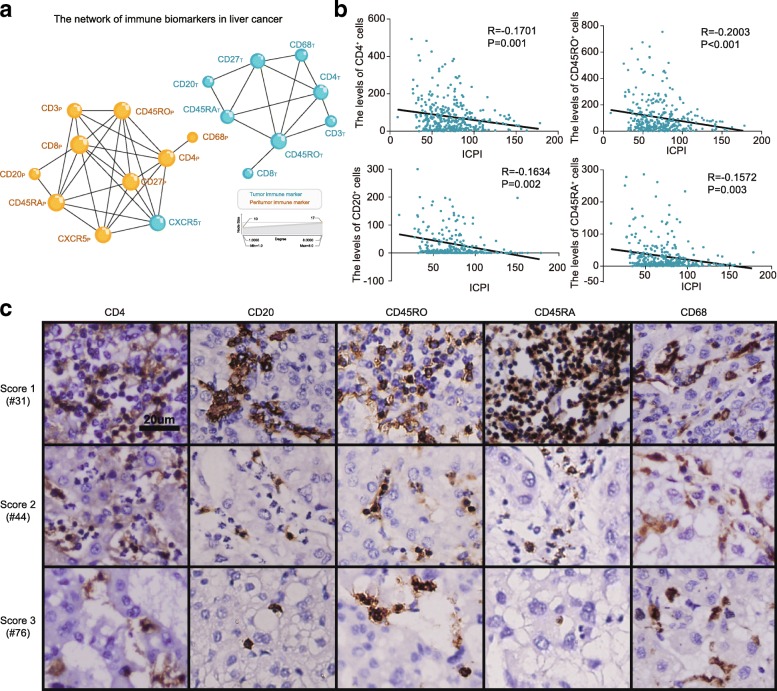
The relationship between ICPI and local immune status. **a** The network of immune features in HCC tissues. **b** Correlation between ICPI and the density of intratumoral CD4^+^, CD20^+^, CD45RO^+^, and CD45RA^+^ cells. **c** Immunostaining of intratumoral CD4^+^, CD20^+^, CD45RO^+^, CD45RA^+^, and CD68 cells across 3 subgroups. Bar, 20 μm

To investigate the interaction of immune features in the tumor microenvironment, an immune network was constructed on the basis of TMA data. Figure 4a shows a clear separation between the two regions, each exhibiting a distinct and characteristic immune cell pattern, and subnetworks of T cell subpopulations (CD3 cells, CD4 cells, memory T cells, and naïve T cells) in liver tissues. We also observed a correlation between B cells (CD20) and the T cell subset network, implying a functional interaction between B cells and T cells. Interestingly, close correlations between memory T cells (CD45RO) and 5 other immune features, including CD3, CD4, CD27, CD45RA, and CD8, existed in both regions. The local interaction of the immune network suggested the existence of tumor-microenvironment compartments with different compositions, which might influence the activity and mobility of T and B lymphocytes in the tumor progression.

The local immune status plays an essential role in carcinogenesis and in response to cancer therapeutics. Previous reports showed that the intratumoral densities of CD8^+^, CD57^+^, and CD45RO^+^ cells correlated with the local immune status [34, 42, 43]. Then, we investigated whether our constructed ICPI could be a potential indicator of the local immune response. The correlations between the ICPI and intratumoral immune features were assessed. Figure 4 b and c reveal that the levels of CD4^+^, CD20^+^, CD45RA^+^, and CD45RO^+^cells were inversely associated with individual ICPI. And also, the relationships between the levels of CD4^+^, CD20^+^, CD45RA^+^, and CD45RO^+^ cells and the TRIS shared similar trends (Additional file 3: Figure S7), implying that the ICPI was a useful prognostic mode and might be correlated with the local immune status.

## Discussion

HCC is a remarkably heterogeneous type of malignancy resulting from the accumulation of epigenetic and genomic alterations and is influenced by tumor-host crosstalk [44, 45]. With inadequate information on the biological behavior and microenvironment characteristics of liver cancer, it may not be accurate enough to simply combine few clinicopathological parameters together for the prediction of survival in HCC patients. In this study, an immune and clinical prognostic index was developed and validated in an independent dataset. The newly developed ICPI was useful in survival prediction for patients with early/intermediate-stage HCC (BCLC stages 0, A, and B).

To identify prognosis-related immune predictors, 28 immune features were evaluated in the training cohort of 352 HCC specimens. In contrast to other studies that used gene expression profiles and flow cytometry, IHC was used for the identification of tumor-infiltrating immune cells, because of its ease of use and clinically applicable. Based on all the examination results, five immune features were selected through LASSO Cox method in our study, including CD3_T_, CD27_T_, CD103_T_, PD1_T_, and CD68_P_. This selection is more comprehensive than those previously reported studies in multiple aspects [25, 46]. First, we systematically investigated 28 immunological features based on intra- and peri-tumoral tissues in liver cancer that are not confined to the commonly used CD3, CD4, CD45RO, or CD8. Second, the LASSO Cox regression model was adopted to achieve covariate selection. Also, in contrast to previous studies with relatively smaller sample sizes (from 65 to 348 patients) [25, 26, 46], our study recruited a much larger cohort (*n* = 745 patients).

The prognosis values of the five immune features selected for TRIS were consistent with previous studies. Our study indicated that a low density of intratumoral CD3^+^ cells predicted poor prognosis, which is similar to studies on HCC [25] and gastric cancer [34]. Consistent with the study by Garnelo et al. [8], high density of CD27-positive cells was associated with superior HCC patients’ survival in our study. Also, we confirmed the interaction between intratumoral B (CD20) cells and T (CD27) cells with immune cell network. In tumor-infiltrating lymphocytes and peripheral blood of HCCs, Zhang et al. [32] revealed that the prevalence of Tregs expressing high levels of HLA-DR, GITR, and CD103 increased during the progressive stages. In accordance with our previous study [47], we observed that high infiltration of PD1^+^ immune cells could predict dismal survival in HCC patients. Furthermore, it has also been reported that Kupffer cells (CD68), the resident macrophages in the liver, play an important role in HCC development and hepatic homeostasis [48]. Similar to the previous study by Zhu et al. [23], high levels of peritumoral macrophages were correlated with tumor progression and poor prognosis after liver resection, indicating the potential value of peritumoral immune components to serve as a prognosis factor and future target of immunotherapy.

Apart from the selected immune biomarkers of CD3, CD27, CD103, CD68, and PD1, the status and prognostic significance of other immune biomarkers were also evaluated in this study. In HCC, the antitumor effector functions of CD4^+^ T and CD8^+^ T cells have been reported [24], while the role of B lymphocytes in the carcinogenesis of HCC remains controversial [4]. The antitumor function of NK cells has been well-documented, which is gradually diminished with the progression of HCC. In our study, no significant differences in NK cells (CD57) were observed between intra- and peri-tumoral tissues. In our previous study, intratumoral neutrophils, facilitating tumorigenesis and resistance to the tyrosine-kinase inhibitor sorafenib, were reported as a poor indicator for HCC patients [28]. Similar to the studies on colorectal cancer [16], the intratumoral memory T cells (CD45RO) were also decreased dramatically in our study. In addition, depletion of CD14^+^ and CD45RA^+^ cells in the tumor regions was observed. However, the intratumoral CXCR5^+^ cells did not show any significant changes.

The host immunity against tumors is considered increasingly important in clinical practice and cancer research as the local immune status can influence tumor evolution. Changes in density of intratumoral-infiltrated immune cells during tumor invasion and metastasis may indicate progressive immune escape. In our study, the ICPI was inversely but weakly correlated with CD4^+^, CD45RA^+^, CD45RO^+^, and CD20^+^cells. Previous studies have reported that the intratumoral immune response was associated with the densities of localized CD3^+^, CD8^+^, and CD45RO^+^ cells [49]. Garnelo et al. reported that tumor-infiltrating CD20^+^ and CD27^+^ cells enhanced local immune activation and contributed to a better prognosis for patients with HCC [8]. In addition, CD103^+^ cells have been proposed as an indicator of the favorable OS in breast and lung cancers [50, 51]. These findings suggest that the combination of selected markers in the ICPI may serve as a potential indicator which represents the localized immune status in liver cancer.

The survival of HCC patients depends on the tumor stage, underlying liver function, and performance status. As a marker of liver injury, GGT was an independent prognostic predictor on multivariate analysis in our study. Recently, other researchers reported that elevated serum GGT was associated with the risk of cancer, especially in liver cancer [52]. Our final ICPI model also integrated tumor diameter and histologic differentiation as reported in previous studies [53, 54]. Based on these clinical predictors, our established ICPI integrated the characteristics of liver function, liver pathology, and host-tumor interaction.

Our study has limitations. First, this study was focused on HBV-associated HCC in the early/intermediate stage. It requires further study whether the ICPI can be applied to patients with advanced-staged disease or HCC with other etiologies requires further studies. Second, the study is retrospective with its inherent defects. Third, the study cohorts were recruited from one single center, and approximately 10% of HCC patients at intermediate stage (BCLC B) underwent surgical resection, which might result in selection bias. In addition, the biological mechanisms remain to be further investigated about how the candidate markers, including CD3, CD27, CD68, CD103, and PD1, are involved in HCC. Finally, the ICPI model was constructed on the basis of pre- and postoperative parameters, which may not be suitable for preoperative decision-making.

## Conclusion

In summary, the ICPI improved the accuracy of survival prediction for patients with early/intermediate-stage HCC. Prospective studies are needed to further validate its analytical accuracy in estimating prognosis for individualized management of HCC patients.

## Additional files

Additional file 1: Supplementary methods. (DOCX 40 kb)

Additional file 2: **Table S1.** The relationship between TRIS and clinicopathological variables. **Table S2.** Comparison of prognostic performance among postoperative variables and ICPI. **Table S3.** Comparison of prognostic performance among HCC staging systems and ICPI. Table S4. Components of 7 staging systems for hepatocellular carcinoma. Table S5. Antibody sources and staining conditions. (DOCX 28 kb)

Additional file 3: **Figure S1.** Study flowchart. **Figure S2.** Digital image analyzed using the image software (Image-pro plus 6.0), with tissue represented in yellow and stained cells represented in red. **Figure S3.** Immunohistochemistry expression pattern of 14 immune infiltrations in tumor and adjacent liver tissues, including CD3, CD4, CD8, CD14, CD20, CD27, CD45RA, CD45RO, CD57, CD66b, CD68, CD103, CXCR5, and PD1. **Figure S4.** X-tile plots of ICPI in the training cohort automatically selecting the optimum cut point according to the highest *χ*^2^ value defined by the Kaplan-Meier survival analysis and log-rank test. **Figure S5.** Kaplan-Meier survival curves of the training cohort categorized by different staging systems. **Figure S6.** (A) Nomogram for predicting the survival probability in HCC patients. (B) Calibration of the predictive models at 1, 3, and 5 years in the derivation and validation cohorts. **Figure S7.** The correlation between TRIS and the density of intratumoral immune features, including CD4^+^, CD20^+^, CD45RO^+^, and CD45RA^+^ cells. (DOCX 5565 kb)

## Abbreviations

AFP: Alpha fetoprotein
AIC: Akaike information criterion
AJCC: American Joint Committee on Cancer
BCLC: Barcelona Clinic Liver Cancer
CA19-9: Carbohydrate antigen 19-9
CEA: Carcinoembryonic antigen
CI: Confidence interval
CLIP: Cancer of the Liver Italian Program
DC: Dendritic cell
GEO: Gene Expression Omnibus
GGT: γ-Glutamyl transferase
HCC: Hepatocellular carcinoma
ICPI: Immune-clinical prognostic index
IHC: Immunohistochemistry
JIS: Japan Integrated Staging
LASSO: Least absolute shrinkage and selection operator
LCSGJ: Liver Cancer Study Group of Japan
NK: Natural killer
OS: Overall survival
RFS: Recurrence-free survival
ROC: Receiver operating characteristic curve
TCGA: The Cancer Genome Atlas
Tfh: T follicular helper
Treg: T regulatory cell
TRIS: Tissue-related immune signature
