# Supplementary material for: Tissue-infiltrating lymphocytes signature predicts survival in patients with early/intermediate stage hepatocellular carcinoma
Source: BMC Med. 2019 Jun 5;17:106. doi: 10.1186/s12916-019-1341-6 (PMC6549297; doi:10.1186/s12916-019-1341-6)
Supplement: Supplementary file 1 — Supplementary methods. (DOCX 40 kb) [file 12916_2019_1341_MOESM1_ESM.docx]

**Additional file**

**Additional file 1: Supplementary methods**

***Immune Cell Fractions Constructed by CIBERSORT Algorithm***

Samples profiled on Illumina or Agilent platforms were downloaded as normalized matrices from public repositories (either NCBI or TCGA), and probes were converted to HUGO gene symbols using chipset definition files available from the NCBI GEO datasets. The TCGA and 7 GEO datasets (GSE10143, GSE14520, GSE25097, GSE39791, GSE54236, GSE63898 and GSE76427) were included in the present study.

The detailed procedures of CIBERSORT could be followed by the CIBERSRT manual online ([https://cibersort.stanford.edu/manual](https://cibersort.stanford.edu/manual.php#sigfile)). Briefly, the 22 cell types inferred by CIBERSORT encompass natural killer cells, T cells, macrophages, B cells, dendritic cells, eosinophils, and neutrophils and so firth [1]. Gene expression datasets were prepared using standard annotation files and data uploaded to the CIBERSORT web portal (<https://cibersort.stanford.edu/>). Deconvolution was achieved with the standard LM22 signature gene file and 1000 permutations to calculate deconvolution *P* values [2].

***Establishment of Immune Network***

We accumulated and preprocessed the data from IHC and CIBERSORT analysis of TCGA or GEO datasets to construct the immune network. The construction and analysis were based on WGCNA [3], which is a typical algorithm. In the immune network, the nodes represent the immune cells and the lines indicate the relevance of the immune cells. In the WGCNA algorithm, the elements in the co-expression matrix are defined as the weighted value of the correlation coefficient. The network was built based on the connectivity between nodes. The selection for the weighted value is that network could satisfies the scale-free law [4]. We got 28 immune features derived from IHC evaluation and 44 immune fractions from CIBERSORT analysis, respectively. The Pearson correlation coefficient between modules was used to construct the network. When the absolute value of correlation was more than 0.5, we would link two immune cells and the network was constructed [5].

***Categorization of Patients in Staging Systems***

The patients were categorized according to 7 current staging systems: Okuda, BCLC, the Cancer of the Liver Italian Program (CLIP) score, Japan Integrated Staging (JIS) score, the 7^th^ and 8^th^ American Joint Committee on Cancer (AJCC) and the Liver Cancer Study Group of Japan (LCSGJ) staging systems (**Table S4**).

***Immunohistochemistry (IHC)***

IHC procedure was described previously [5-7]. Briefly, before proceeding with staining procedure, the slides of tissue microarray (TMA) were deparaffinized and rehydrated. Endogenous peroxidase activity was blocked in 3% H_2_O_2_. The antigens of 14 proteins were retrieved at 98°C for 30min with 10mM citrate buffer (pH 6.0) or 1mM EDTA buffer (pH 7.5). Then, TMA were cooled and put in PBS solution. After blocking in 5% bovine serum albumin (BSA) for 1h at room temperature, arrays were blotted and covered with each of the antibodies to the 14 proteins and incubated at 4°C overnight. Arrays were then washed with PBS. Slides were placed in the detection system of Histostain^®^–Plus Kits (Invitrogen Corporation) and DAB following counterstaining with hematoxylin. Blank controls were treated identically except that primary antibodies were omitted. The expression pattern, cellular localization, species, staining conditions of 14 immune antibodies, and biomarker-correlated immune cells are summarized in **Table S5**.

**References**

1. Ali HR, Chlon L, Pharoah PD, Markowetz F, Caldas C: **Patterns of Immune Infiltration in Breast Cancer and Their Clinical Implications: A Gene-Expression-Based Retrospective Study**. *PLoS medicine* 2016, **13**(12):e1002194.

2. Newman AM, Liu CL, Green MR, Gentles AJ, Feng W, Xu Y, Hoang CD, Diehn M, Alizadeh AA: **Robust enumeration of cell subsets from tissue expression profiles**. *Nature methods* 2015, **12**(5):453-457.

3. Luo Y, Coskun V, Liang A, Yu J, Cheng L, Ge W, Shi Z, Zhang K, Li C, Cui Y *et al*: **Single-cell transcriptome analyses reveal signals to activate dormant neural stem cells**. *Cell* 2015, **161**(5):1175-1186.

4. Barabasi AL: **Scale-free networks: a decade and beyond**. *Science (New York, NY)* 2009, **325**(5939):412-413.

5. Zhou SL, Dai Z, Zhou ZJ, Chen Q, Wang Z, Xiao YS, Hu ZQ, Huang XY, Yang GH, Shi YH *et al*: **CXCL5 contributes to tumor metastasis and recurrence of intrahepatic cholangiocarcinoma by recruiting infiltrative intratumoral neutrophils**. *Carcinogenesis* 2014, **35**(3):597-605.

6. Brunner SM, Rubner C, Kesselring R, Martin M, Griesshammer E, Ruemmele P, Stempfl T, Teufel A, Schlitt HJ, Fichtner-Feigl S: **Tumor-infiltrating, interleukin-33-producing effector-memory CD8(+) T cells in resected hepatocellular carcinoma prolong patient survival**. *Hepatology (Baltimore, Md)* 2015, **61**(6):1957-1967.

7. Li YW, Qiu SJ, Fan J, Zhou J, Gao Q, Xiao YS, Xu YF: **Intratumoral neutrophils: a poor prognostic factor for hepatocellular carcinoma following resection**. *Journal of hepatology* 2011, **54**(3):497-505.
