# Supplementary material for: Tissue-infiltrating lymphocytes signature predicts survival in patients with early/intermediate stage hepatocellular carcinoma
Source: BMC Med. 2019 Jun 5;17:106. doi: 10.1186/s12916-019-1341-6 (PMC6549297; doi:10.1186/s12916-019-1341-6)
Supplement: Supplementary file 2 — Table S1. The relationship between TRIS and clinicopathological variables. Table S2. Comparison of prognostic performance among postoperative variables and ICPI. Table S3. Comparison of prognostic performance among HCC staging systems and ICPI. Table S4. Components of 7 staging systems for hepatocellular carcinoma. Table S5. Antibody sources and staining conditions. (DOCX 28 kb) [file 12916_2019_1341_MOESM2_ESM.docx]

**Additional file 2:**

**Table S1. The relationship between TRIS and clinicopathological variables (n=352).**

| Categorical variables | n (%) | *P* value |
| --- | --- | --- |
| Sex (female) | 60 (17.0%) | 0.02 |
|  | | |
| Continuous variables | *β*-se | *P* value |
| AFP, ng/ml | 9639.061 (3668.562） | 0.009 |
| Tumor numbers, n (%) | -0.379 (0.190) | 0.046 |
| Tumor diameter, cm | 7.617 (3.343) | 0.02 |

**Table S2. Comparison of prognostic performance among postoperative variables and ICPI.**

| Variables | Training (n=352) | | Validation (n=393) | |
| --- | --- | --- | --- | --- |
|  | C-index (95%CI) | *P* value | C-index (95%CI) | *P* value |
| ICPI | 0.691 (0.642, 0.739) |  | 0.686 (0.637, 0.735) |  |
| Microvascular invasion | 0.573 (0.535, 0.610) | <0.001 | 0.585 (0.548, 0.622) | <0.001 |
| Lymphoid metastasis | 0.502 (0.497, 0.508) | <0.001 | 0.504 (0.496, 0.512) | <0.001 |
| Tumor differentiation | 0.564 (0.530, 0.598) | <0.001 | 0.547 (0.510, 0.584) | <0.001 |

**Table S3. Comparison of prognostic performance among HCC staging systems and ICPI.**

| Model | Training cohort (n=352) | | Validation cohort (n=393) | |
| --- | --- | --- | --- | --- |
|  | C-index (95%CI) | *P* value | C-index (95%CI) | *P* value |
| ICPI | 0.691 (0.642, 0.739) |  | 0.686 (0.637, 0.735) |  |
| AJCC 7^th^ for HCC | 0.595 (0.555, 0.635) | 0.008 | 0.519 (0.487, 0.550) | <0.001 |
| AJCC 8^th^ for HCC | 0.589 (0.550, 0.628) | 0.002 | 0.573 (0.535, 0.611) | <0.001 |
| LCSGJ | 0.557 (0.521, 0.592) | <0.001 | 0.553 (0.515, 0.591) | <0.001 |
| BCLC | 0.552 (0.516, 0.589) | <0.001 | 0.544 (0.509, 0.579) | <0.001 |
| JIS | 0.553 (0.518, 0.588) | <0.001 | 0.556 (0.518, 0.594) | <0.001 |
| Okuda | 0.548 (0.522, 0.573) | <0.001 | 0.602 (0.575, 0.630) | <0.001 |
| CLIP | 0.597 (0.555, 0.640) | <0.001 | 0.610 (0.570, 0.650) | 0.01 |

**Table S4. Components of 7 staging systems for hepatocellular carcinoma.**

| Staging systems | Liver function | Performance status (Symptoms) | AFP | Tumor status | | | |
| --- | --- | --- | --- | --- | --- | --- | --- |
|  |  |  |  | Number | Size | Vascular invasion | Metastasis |
| BCLC | CTP class | Performance status | No | Yes | Yes | Yes | Yes |
| CLIP | CTP class | Performance status | Yes | Yes | Yes | Yes | Yes |
| JIS | CTP class | No | No | Yes | Yes | Yes | Yes |
| AJCC 7^th^ | No | No | No | Yes | Yes | Yes | Yes |
| AJCC 8^th^ | No | No | No | Yes | Yes | Yes | Yes |
| LCSGJ | No | No | No | Yes | Yes | Yes | Yes |
| Okuda | Ascites, ALB, TB | No | No | No | Yes | Yes | Yes |

AFP, alpha-fetoprotein; AJCC, American Joint Committee on Cancer; BCLC, Barcelona Clínic Liver Cancer; CLIP, Cancer of the Liver Italian Program; CTP, Child-Turcotte-Pugh; JIS, Japan Integrated Staging; LCSGJ, Liver Cancer Study Group of Japan.

**Table S5. Antibody sources and staining conditions.**

| Markers | Immune cells | Antibody source | Cellular localization | Species | Antigen Retrieval | Dilution |
| --- | --- | --- | --- | --- | --- | --- |
| CD3 | pan T cells | DAKO | Membranous | Mouse Monoclonal | Citrate buffer (pH 6.0) | 1:200 |
| CD4 | CD4 T cells | DAKO | Membranous | Mouse Monoclonal | Tris/EDTA buffer (pH 9.0) | 1:400 |
| CD8 | cytotoxic T cells | Abcam | Membranous | Mouse Monoclonal | Tris/EDTA buffer (pH 9.0) | 1:500 |
| CD14 | mononuclear cells | Sigma | Cytoplasmic | Rabbit polyclonal | Citrate buffer (pH 6.0) | 1:500 |
| CD20 | B cells | Abcam | Membranous | Rabbit polyclonal | Tris/EDTA buffer (pH 9.0) | 1:200 |
| CD27 | plasma cells | Abcam | Membranous | Rabbit Monoclonal | Tris/EDTA buffer (pH 9.0) | 1:500 |
| CD45RA | naive T cells | Abcam | Membranous | Mouse Monoclonal | Citrate buffer (pH 6.0) | 1:200 |
| CD45RO | memory T cells | Thermo Fisher Scientific | Membranous | Mouse Monoclonal | Citrate buffer (pH 6.0) | 1:200 |
| CD57 | NK cells | DAKO | Cytoplasmic | Mouse Monoclonal | Citrate buffer (pH 6.0) | 1:200 |
| CD66b | neutrophils | BD | Cytoplasmic | Mouse Monoclonal | Citrate buffer (pH 6.0) | 1:200 |
| CD68 | macrophages | DAKO | Cytoplasmic | Mouse Monoclonal | Citrate buffer (pH 6.0) | 1:200 |
| CD103 | Tregs | Abcam | Cytoplasmic | Rabbit Monoclonal | Tris/EDTA buffer (pH 9.0) | 1:1000 |
| CXCR5 | Tfh cells | CST | Membranous | Rabbit Monoclonal | Citrate buffer (pH 6.0) | 1:200 |
| PD1 | T cells | CST | Membranous | Rabbit Monoclonal | Citrate buffer (pH 6.0) | 1:200 |
