# Supplementary material for: Tissue-infiltrating lymphocytes signature predicts survival in patients with early/intermediate stage hepatocellular carcinoma
Source: BMC Med. 2019 Jun 5;17:106. doi: 10.1186/s12916-019-1341-6 (PMC6549297; doi:10.1186/s12916-019-1341-6)

**Additional file 3:**

**Fig S1.** Study flowchart. HCC, hepatocellular carcinoma; LASSO, Least Absolute Shrinkage and Selector Operation; ICPI, immune-clinical prognostic index.


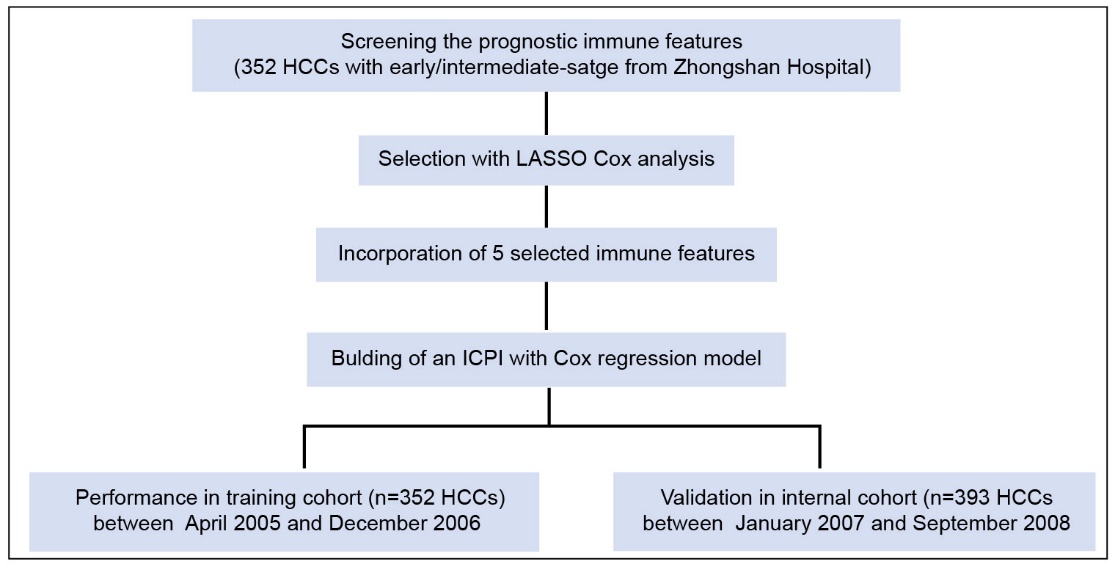


**Fig S2.** Digital image analyzed using the image software (Image-pro plus 6.0), with tissue represented in yellow and stained cells represented in red. Bar, 100 μm.


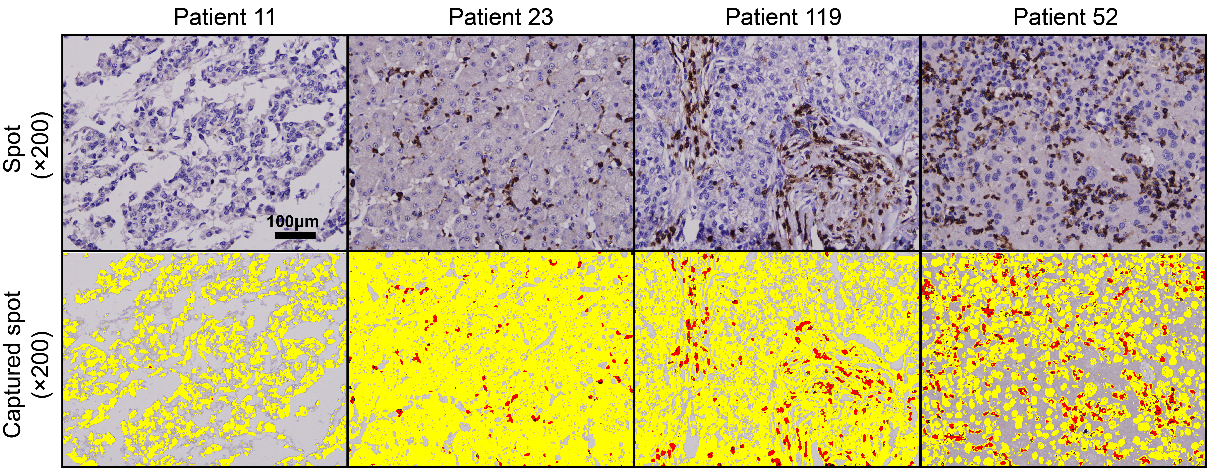


**Fig S3.** Immunohistochemistry expression pattern of 14 immune infiltrations in tumor and adjacent liver tissues, including CD3, CD4, CD8, CD14, CD20, CD27, CD45RA, CD45RO, CD57, CD66b, CD68, CD103, CXCR5 and PD1. Bar, 20 μm.


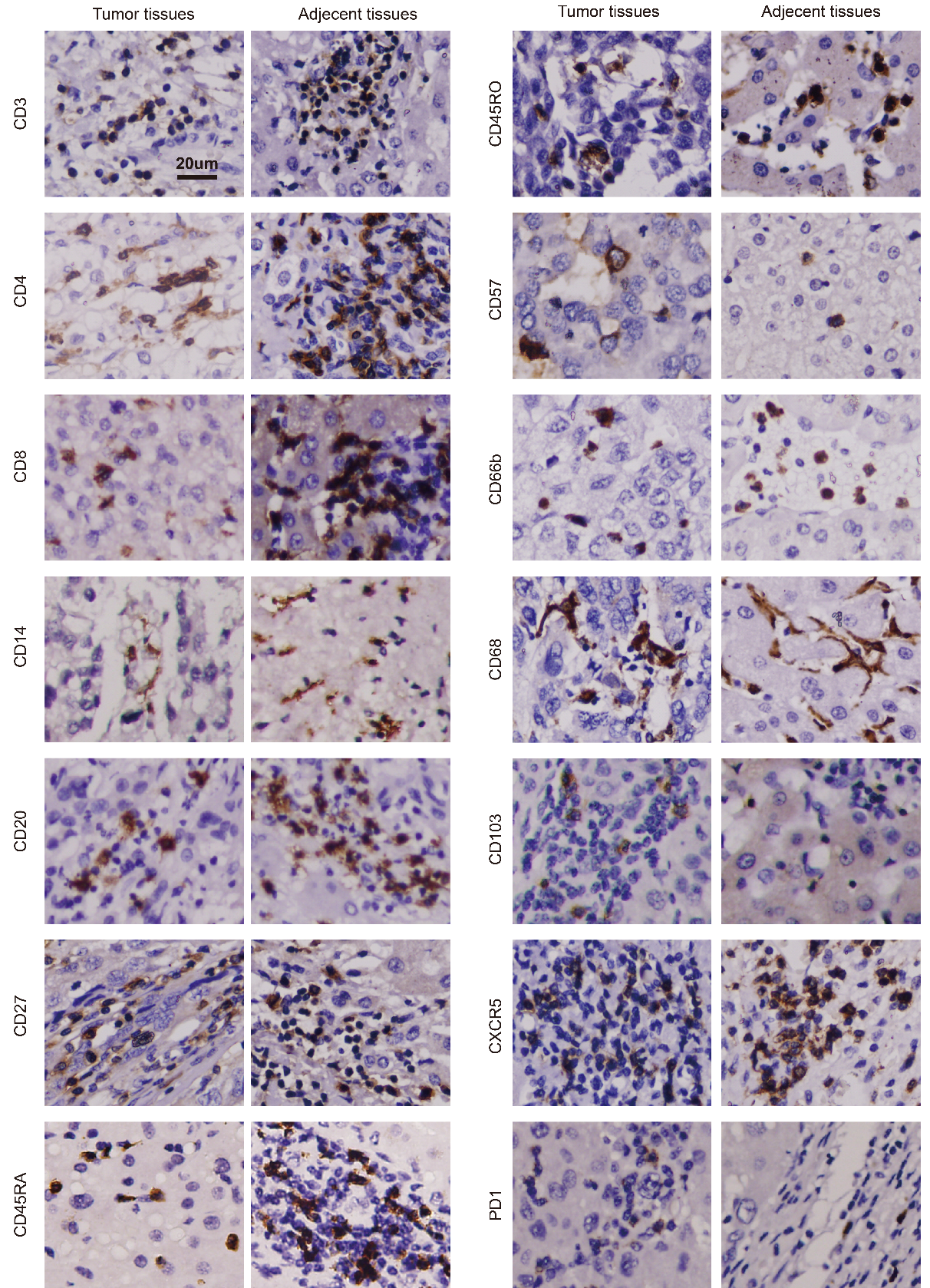


**Fig S4.** X-tile plots of ICPI in the training cohort automatically selecting the optimum cut point according to the highest χ^2^-value defined by Kaplan-Meier survival analysis and log-rank test.


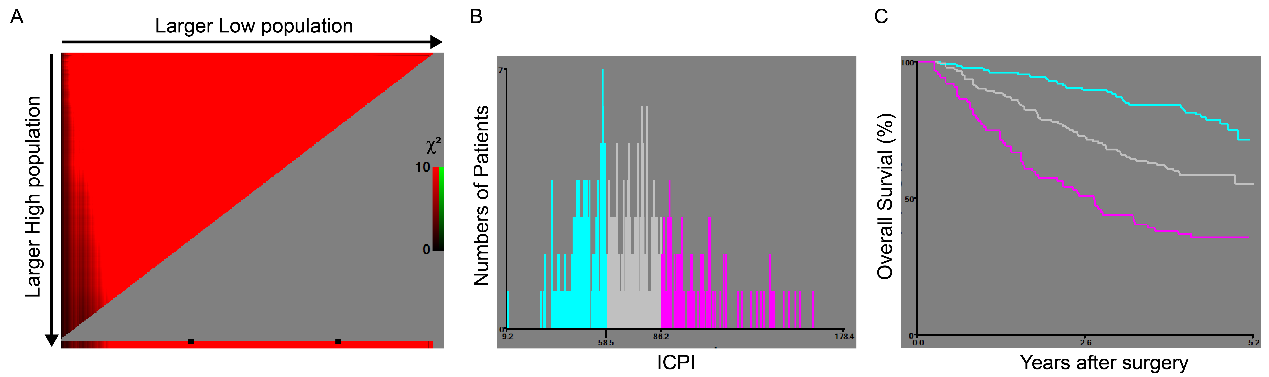


**Fig S5.** Kaplan-Meier survival curves of the training cohort categorized by different staging systems [(A) Stratified ICPI; (B) BCLC; (C) CLIP; (D) JIS; (E) LCSGJ; (F) Okuda; (G) AJCC 7^th^ edition; (H) AJCC 8^th^ edition)].


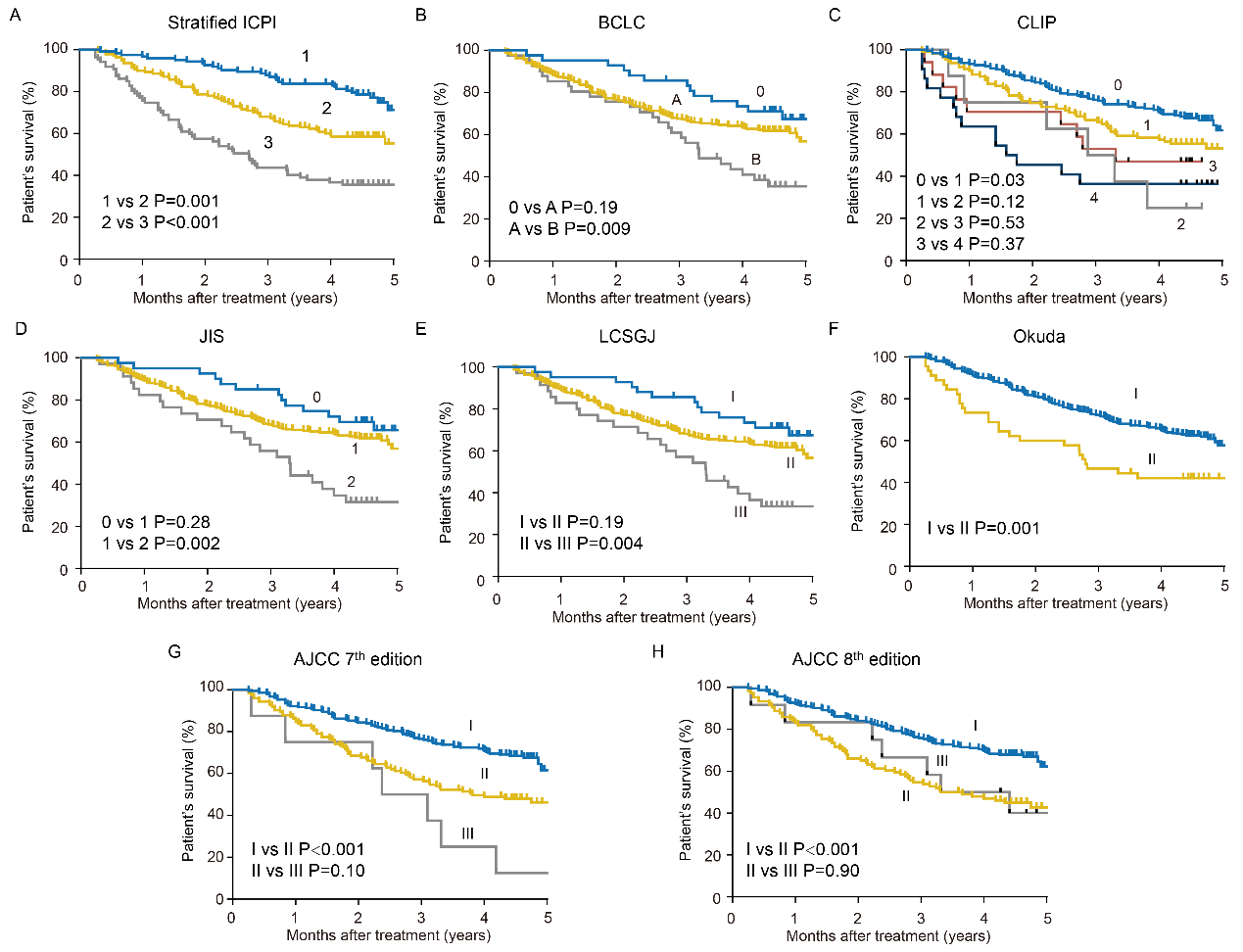


**Fig S6.** (A) Nomogram for predicting the survival probability in HCC patients. (B) Calibration of the predictive models at 1-, 3- and 5- year in the derivation and validation cohorts. Nomogram-predicted probability of overall survival (OS) is plotted on the x-axis; actual OS is plotted on the y-axis.


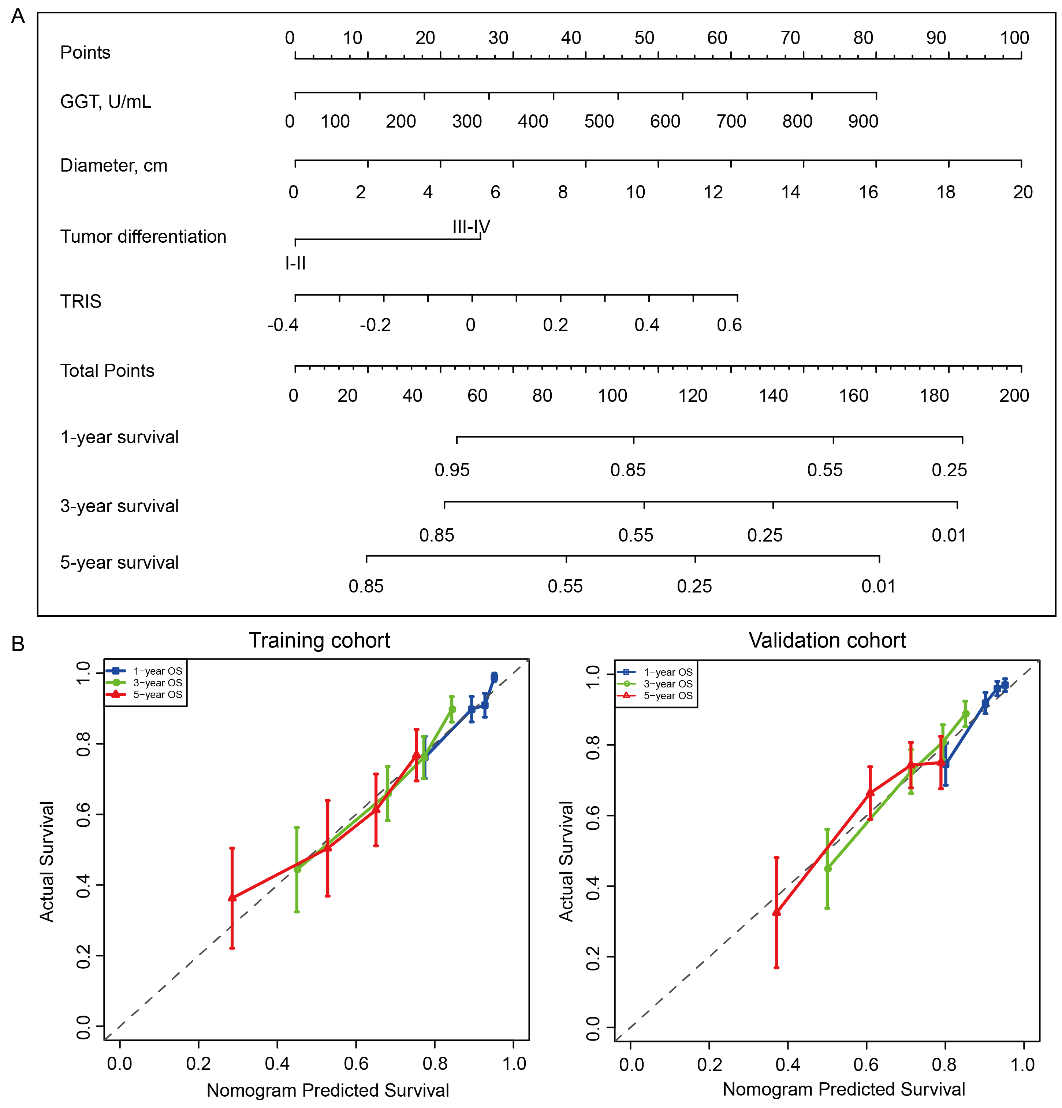


**Fig S7.** The correlation between TRIS and the density of intratumoral immune features, including CD4^+^, CD20^+^, CD45RO^+^, and CD45RA^+^ cells.


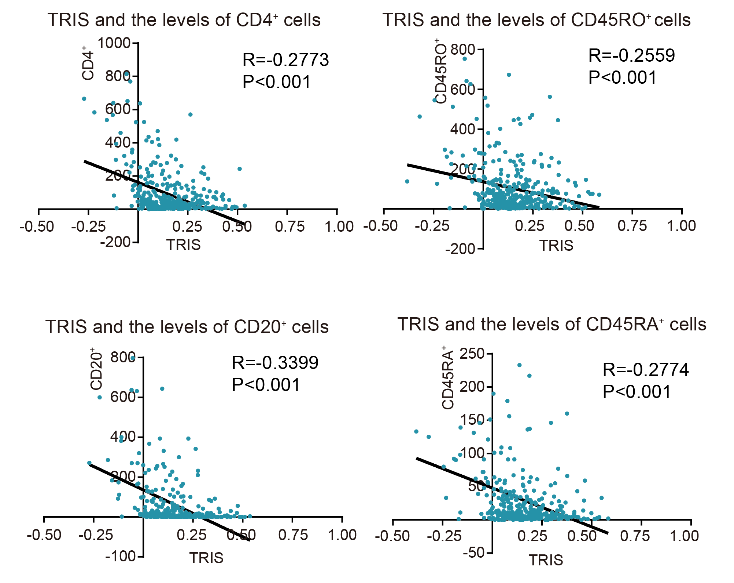

Supplement: Supplementary file 3 — Figure S1. Study flowchart. Figure S2. Digital image analyzed using the image software (Image-pro plus 6.0), with tissue represented in yellow and stained cells represented in red. Figure S3. Immunohistochemistry expression pattern of 14 immune infiltrations in tumor and adjacent liver tissues, including CD3, CD4, CD8, CD14, CD20, CD27, CD45RA, CD45RO, CD57, CD66b, CD68, CD103, CXCR5, and PD1. Figure S4. X-tile plots of ICPI in the training cohort automatically selecting the optimum cut point according to the highest χ2 value defined by the Kaplan-Meier survival analysis and log-rank test. Figure S5. Kaplan-Meier survival curves of the training cohort categorized by different staging systems. Figure S6. (A) Nomogram for predicting the survival probability in HCC patients. (B) Calibration of the predictive models at 1, 3, and 5 years in the derivation and validation cohorts. Figure S7. The correlation between TRIS and the density of intratumoral immune features, including CD4+, CD20+, CD45RO+, and CD45RA+ cells. (DOCX 5565 kb) [file 12916_2019_1341_MOESM3_ESM.docx]
